# Unraveling the Role of Sex Hormones on Keratinocyte Functions in Human Inflammatory Skin Diseases

**DOI:** 10.3390/ijms23063132

**Published:** 2022-03-15

**Authors:** Rossella Gratton, Cecilia Del Vecchio, Luisa Zupin, Sergio Crovella

**Affiliations:** 1 Department of Advanced Diagnostics, Institute for Maternal and Child Health—IRCCS Burlo Garofolo, 34137 Trieste, Italy; 2 Dermatology Unit, Fondazione IRCCS Ca’ Granda Ospedale Maggiore Policlinico, 20122 Milan, Italy; ceciliadelvecchio2@gmail.com; 3 Maternal-Neonatal Department, Institute for Maternal and Child Health—IRCCS Burlo Garofolo, 34137 Trieste, Italy; luisa.zupin@burlo.trieste.it; 4 Biological Sciences Program, Department of Biological and Environmental Sciences, College of Arts and Sciences, University of Qatar, Doha 2713, Qatar; sgrovella@qu.edu.qa

**Keywords:** keratinocytes, sex hormones, inflammatory skin diseases

## Abstract

The skin exerts several fundamental functions that are the first physical, chemical and immune barriers to the human body. Keratinocytes, the main cell type of the epidermis, provide mechanical defense, support skin integrity and actively endorse cutaneous immune responses. Not surprisingly, considering these crucial activities, alterations in keratinocyte functions are associated with different inflammatory skin diseases. Recent findings indicate that the skin should not only be regarded as a target for hormones but that it should also be considered as an endocrine peripheral organ that is directly involved in the synthesis and metabolism of these chemical messengers. Sex hormones have multiple effects on the skin, attributed to the binding with intracellular receptors expressed by different skin cell populations, including keratinocytes, that activate downstream signaling routes that modulate specific cellular functions and activities. This review is aimed at reorganizing the current knowledge on the role exerted by sex hormones on keratinocyte function in five different inflammatory skin diseases: Hidradenitis suppurativa; Acne vulgaris; Atopic dermatitis; progesterone hypersensitivity; psoriasis. The results of our work aim to provide a deeper insight into common cellular mechanisms and molecular effectors that might constitute putative targets to address for the development of specific therapeutic interventions.

## 1. Introduction

The skin possesses a well defined tripartite structure that, from the uttermost to the deepest layer, is provided by the epidermis, the dermis and the hypoderm. This complex and intricate structural organization is strictly required for the accomplishment of a wide variety of activities including [1,2]: immune and endocrine functions; constitution of an initial barrier against pathogens and other environmental noxae, mechanical injury, chemicals and UV radiations; thermoregulatory functions and water retention activities to limit evaporation. 

Keratinocytes are the principal cell type of the epidermis, and their primary activity includes the maintenance of the functional, structural and physical integrity of the skin. Nevertheless, over recent years, consistent evidence revealed that keratinocytes play supplementary roles that go beyond providing a sole protective barrier against external agents. Indeed, upon stimulation, these cells are known to actively participate in the initiation and regulation of cutaneous inflammatory reactions by synthesizing, releasing and responding to different inflammatory, immunomodulatory and immunosuppressive mediators [3]. Moreover, it is well established that keratinocytes are involved both in the production, activation and inactivation of hormones and in responding to circulating hormones that primarily influence differentiation and proliferation programs [1,3,4]. 

In this intriguing context, a thorough characterization of the mechanisms underlying the complex interplay existing between defects in functional and immune properties of keratinocytes, cutaneous immune dysregulation and endocrine alterations should be further investigated since they may be closely associated with a continuously expanding spectrum of human immune-mediated skin diseases [5]. Therefore, this review aims at reorganizing the current knowledge on the role exerted by hormones, specifically of sex hormones, on keratinocyte function in different inflammatory skin diseases, with the aim of providing a deeper insight into common cellular mechanisms and molecular effectors that might constitute putative targets to address for the development of effective therapeutic interventions.

### 1.1. The Skin 

The physical and functional integrity of the skin is tightly linked to its specific structural partition into three layers [3,6]: the epidermis, the uppermost portion defined as a squamous stratified epithelium composed predominantly by keratinocytes and also by dendritic cells (DCs); the dermis, the middle layer bearing a matrix of amorphous connective tissue and collagen, a nervous and vascular network, resident fibroblasts, mast cells and macrophages, and the epidermal appendages (hair follicles, sebaceous glands and apocrine glands); the hypoderm, the deepest level represented by a subcutaneous vascularized tissue constituted by lobules of adipocytes separated by collagen fibres.

Specifically, the epidermis is composed of the interfollicular epidermis (IFE) and associated skin appendages, collectively organized to form the principal interface between the external environment and the host. The major functions of the epidermis encompass [6]: innate immunity activities through the constitution of a protective barrier acting against pathogens and other environmental agents; endocrine activities due to the ability to produce hormones and to respond to the circulating ones; lubrication of the skin through lipid synthesis; constitution of a barrier aimed at limiting transepidermal water loss; thermoregulatory activities induced by the presence of hair and sweat.

**Figure 1:**
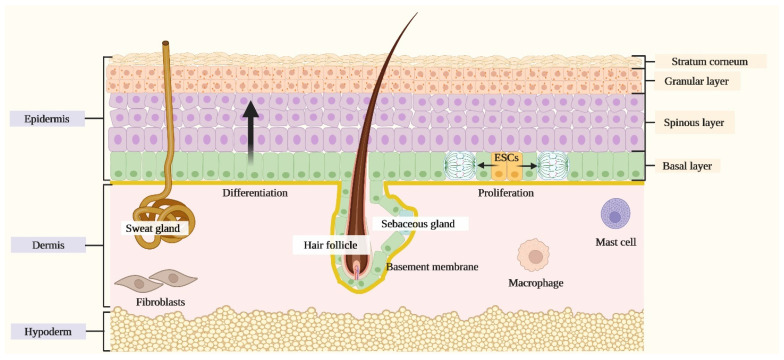
**Schematic representation of the skin.** The skin is composed of three layers: the epidermis, the dermis and the hypoderm. The epidermis, the uppermost portion, is composed of a stratified squamous epithelium comprising the interfollicular epidermis (IFE) and associated skin appendages, including hair follicles, sebaceous glands and apocrine glands. The IFE is formed by progressively differentiating keratinocytes organized in different layers that, from the deepest to most superficial one, comprise the basal layer, the spinous layer, the granular layer and the stratum corneum. The dermis, the middle layer, comprehends a matrix of amorphous connective tissue and collagen, a nervous and vascular network, resident fibroblasts, mast cells and macrophages. The hypoderm, the deepest level, is organized into a subcutaneous vascularized tissue constituted by lobules of adipocytes separated by collagen fibres.

The IFE is composed both by actively proliferating and progressively differentiating keratinocytes that are arranged into four layers that, from the deepest to the most superficial one, compose the basal layer, the spinous layer, the granular layer and the stratum corneum [3]. The mitotically active cells located in the basal layer and resting on the basement membrane, namely epidermal stem cells, generate either other stem cells that self-renew or transient amplifying cells at every division cycle. The latter is defined as a population that stops proliferating and progressively undergoes terminal differentiation through a process that is regulated by specific local signals that drive keratinocytes to migrate upwards towards the stratum corneum and to sequentially express layer-specific components including epidermal keratins [3] (Figure 1).

In this district, a strict synergistic balance between keratinocyte differentiation and proliferation programs is required; indeed, deregulations in these processes are associated with the pathogenesis of various inflammatory skin diseases, including Hidradenitis suppurativa [7], Acne vulgaris [8], Atopic dermatitis [9], and Psoriasis [10]. 

### 1.2. Cutaneous Immune Responses 

The skin possesses a crucial role in host defence by providing a passive protective mechanical barrier that is tightly integrated with active immune surveillance mechanisms aimed at creating a robust and intricate defence network. Cutaneous immune responses are primarily processed by cellular populations that act as sentinels for the recognition of dangerous stimuli and include keratinocytes and resident immune cells encompassing macrophages, dermal DCs, Langerhans cells (LCs) and dermal mast cells [11]. 

Briefly, during the early stages of innate cutaneous immune responses, keratinocytes together with activated LCs, macrophages and dermal DCs are involved in the rapid synthesis and release of pro-inflammatory cytokines, antimicrobial peptides (AMPs) and chemotactic molecules. These responses are primarily mediated by the activation of pathogen recognition receptors (PRRs) that are involved in the recognition of conserved pathogen components, namely the Toll-like receptors (TLRs) and nucleotide-binding oligomerization domain (NOD)-like receptors (NLRs), and by the production of inflammatory and immune molecules that trigger further downstream signalling pathways [5,11]. 

In this context, keratinocytes are well known to orchestrate the initial responses to different cutaneous local stimuli by initiating and regulating the early innate immune responses by producing cytokines including the potent inducer of local immune functions interleukin-1α (IL-1α) and interleukin-1β (IL-1β), tumor necrosis factor (TNF), other cytokines, chemokines and AMPs. Next, the pro-inflammatory and immunomodulatory molecules synthesized and released by keratinocytes activate cutaneous DCs, thus stimulating the expression of other immune mediators that further sustain the development of local inflammatory responses and favor the in situ recruitment of immune cells from the bloodstream [11,12,13].

**Figure 2:**
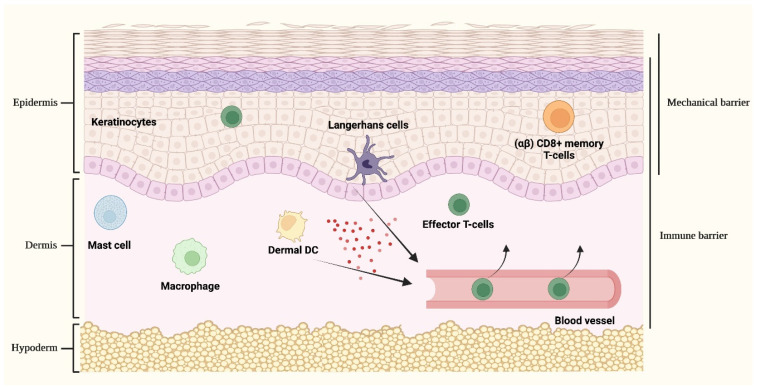
**Cutaneous immune responses.** The skin provides a passive protective mechanical barrier tightly integrated with an active immune barrier, collectively aiming at creating a robust and intricate defence network. The mechanical barrier against external noxae is given by the highly packed keratinocytes of the epidermis. Different cellular populations act as sentinels involved in the recognition of dangerous stimuli and, upon activation, are implicated in orchestrating complex cutaneous immune responses. These cell types include keratinocytes, Langerhans cells and (αβ) CD8+ memory T-cells that are located in the epidermis, dermal dendritic cells (DCs), mast cells and macrophages. Upon stimulation, the activation of keratinocytes and epidermal/dermal resident immune cells leads to the release of cytokines and chemokines (represented by red spheres in the figure) that collectively sustain inflammatory responses and favor the recruitment of activated inflammatory cells from the bloodstream, principally of effector T-cells, hence redirecting the immune response towards the initial cutaneous inflammatory site.

Activated cutaneous DCs, including LCs and dermal DCs, are involved in the priming and initiation of cutaneous adaptive immune responses by prompting both the sensitization and the elicitation processes. Following antigen recognition and engulfment in the skin, during the sensitization phase, activated DCs migrate to skin-draining lymph nodes, where they mature and encounter naïve T cells in order to promote their differentiation in effector T cells that express homing receptors for the skin, hence redirecting the immune response towards the initial cutaneous inflammatory site. Once in the skin, recruited effector T cells start to accumulate both in the dermis and epidermis, sites in which they encounter a wide variety of costimulatory signals, including inflammatory mediators generated by innate immune responses and other activated antigen-presenting DCs, and are stimulated to produce cytokines and chemokines that promote the development of antigen-specific immune responses (elicitation phase) [11,14]. Moreover, also involved in crucial immune surveillance mechanisms are the skin-resident (αβ) CD8+ memory T-cells that have previously undergone activation, clonal expansion and recruitment to the skin following a response to an antecedent antigenic stimulation, thus guaranteeing rapid protection in the case of re-exposure to an already encountered immunogen molecule [15,16] (Figure 2).

#### Role of Keratinocytes in Skin Immunity

The necessity to consider keratinocytes, the most abundant cellular component of the epidermis, not only as passive constituents composing the physical barrier of the skin but also as crucial actors in early cutaneous immune defence and tolerance mechanism is currently widely accepted. Indeed, keratinocytes are considered as the principal initiators of inflammatory processes since these cells can synthesize and respond to a wide variety of cytokines, chemokines and growth factors, which are involved in the direct synthesis of AMPs and express a wide array of PRRs isoforms [15,17]. In addition, keratinocytes are involved in the coordination of complex cross-talks with other immune cells that are essential to allow the maintenance of skin homeostasis, the preservation of defence mechanisms against environmental noxae and the development of immune memory [18,19]. These cellular interactions occur principally either through direct cell-to-cell contact, including antigen presentation to skin-resident (αβ) CD8+ memory T-cells [18] or via secretion of keratinocyte-derived vesicles containing cargoes aimed at regulating various biological processes including proliferation, inflammation, differentiation, pigmentation and wound healing [20]. 

**Figure 3:**
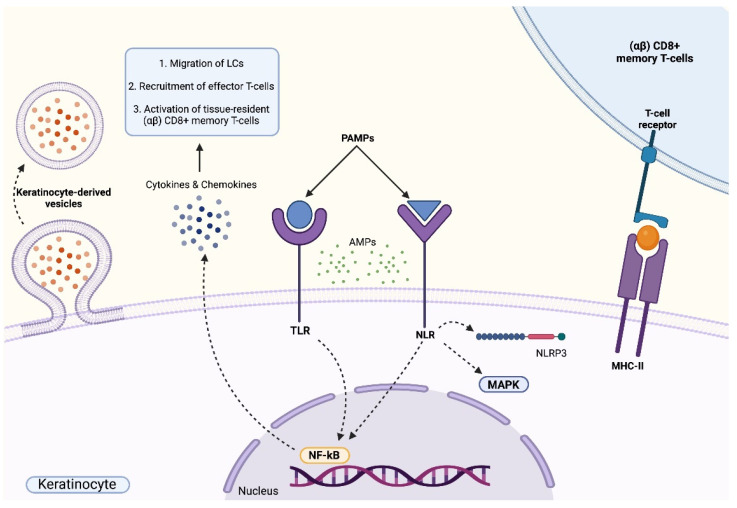
**Immune functions of keratinocytes.** Keratinocytes actively participate in early cutaneous immune defence. Membrane-bound Toll-Like receptors (TLRs) and nucleotide-binding oligomerization domain (NOD)-like receptors (NLRs) recognize pathogen-associated molecular patterns (PAMPs), an event that triggers the activation of intracellular pro-inflammatory signalling cascades. The activation of TLRs leads to the activation of the NF-кB signalling pathway that results in the release of various cytokines and chemokines that sustain inflammatory responses and favor the recruitment of activated inflammatory cells. The stimulation of NLRs is characterized by the production and secretion of pro-inflammatory cytokines due to the activation of the NLR family pyrin domain containing 3 (NLRP3) inflammasome platform and of the MAPK and NF-кB signalling pathways. The activation of TLRs and NLRs induces the release of antimicrobial peptides (AMPs). Keratinocytes are involved in the coordination of complex cross-talks with other immune cells, including antigen presentation to skin-resident (αβ) CD8+ memory T-cells or via secretion of keratinocyte-derived vesicles containing cargoes aimed at regulating various biological processes including proliferation, inflammation, differentiation, pigmentation and wound healing.

The initiation of immune responses in keratinocytes is driven by the recognition of pathogen-associated molecular patterns (PAMPs) to membrane-bound TLRs and NLRs that subsequently induce the activation of pro-inflammatory signalling cascades. Indeed, the activation of TLRs in keratinocytes is usually associated with the induction of pro-inflammatory responses that lead to the production of inflammatory cytokines, including IL-1β, IL-6, IL-8, IL-18 and interferons (INFs), principally, through the activation of the NF-кB pathway; actually, the activation of the NF-кB signalling cascade collectively promotes the migration of LCs to skin-draining lymph nodes and to the recruitment of effector T cells that express homing receptors for the skin. Moreover, the activation of TLRs also stimulates keratinocytes to further release cytokines (e.g., CCL20 and CCL27) and chemokines (e.g., CXCL9 and CXCL10), thus favoring the activation of tissue-resident (αβ) CD8+ memory T-cells [21]. Next, the intracellular transduction routes triggered by the activation of NLRs primarily induce either the activation of the NLR family pyrin domain containing 3 (NLRP3) inflammasome platform; therefore, they promote the production and secretion of pro-inflammatory cytokines including IL-1β and IL-18, or the stimulation of inflammation through the MAPK and NF-кB signalling pathways [15,22]. In addition, upon the activation of PRRs, keratinocytes synthesize and release AMPs, molecules that not only grant the exploitation of direct antimicrobial activities but that are also involved in the maintenance and amplification of inflammatory responses by other cutaneous resident immune cells [15,23] (Figure 3).

Given the fundamental role of keratinocytes in skin immunity, the presence of modifications in their immune properties is associated with the pathophysiology of various inflammatory skin disorders [15]. The impaired ability of keratinocytes to communicate with other immune cells in order to properly coordinate inflammatory reactions need to be further foreseen. A deep characterization of the impact of these aberrant responses in the onset and progression of various immune-mediated skin diseases might shed light on novel cellular and molecular mechanisms that might favor the identification of possible molecular targets to be addressed for the development of new treatments for cutaneous disorders [3,19].

### 1.3. The Skin as a Target of Hormones and as an Independent Peripheral Endocrine Organ

The endocrine system is composed of an intricate and well-organized network of glands and hormone-producing tissues distributed throughout the body whose primary function is the synthesis and secretion of hormones, chemical messengers that are involved in the regulation of a wide spectrum of physiological activities, including growth, developmental and reproductive processes, the maintenance of a stable internal environment by strictly balancing the homeostasis of electrolytes and of nutrient metabolism and the integration of body responses to external stimuli [24,25]. 

In the case of human skin, recent findings strongly indicate the necessity to reconsider the strict interactions between the cutaneous district and hormones. Indeed, the skin is not only regarded as a target site of several hormones that exert well-established functions primarily involved in the regulation of cutaneous development and the maintenance of the homeostasis of the skin tissue [26], but also as a district that is itself directly involved in the synthesis, activation, inactivation and elimination of several hormones and hormone-like molecules possessing systemic, paracrine, autocrine and intracrine activities [4].

#### 1.3.1. The Skin as a Target Site for Hormones

Target districts generally respond to endocrine hormones following hormonal binding to intracellular or to cell surface receptors, interactions that result in the formation of hormone-receptor complexes that trigger the activation of intracellular routes, ultimately leading to the modulation of specific cellular functions and activities [24].

Hormones synthesized by hormone-producing glands exercise well-characterized pleiotropic regulatory effects on different skin cell types due to their expression of high-affinity membrane-bound, cytoplasmic or nuclear hormone-receptors [27].

Located on the cell membrane of various skin cell populations are receptors specialized in the recognition of neurotransmitters and peptide hormones that function by activating hormone-specific second messengers and specific signal transduction routes in target cells [23].

Conversely, cytoplasmic and nuclear hormone receptors, primarily targeted by steroid and thyroid hormones, are defined as soluble molecules that act as transcriptional regulators following the recognition of specific DNA sequences, namely hormone-responsive elements (HRE) [4]. Under basal conditions, steroid receptors are localized in the cytoplasm as polymeric structures tightly associated with heat-shock proteins. Upon binding with the steroid ligand, the receptor undergoes significant conformational changes that induce a dissociation from the heat-shock proteins and the exposition of signal sequences that allow the translocation of the hormone-receptor complex to the nuclear compartment [27]. On the contrary, thyroid hormone receptors are exclusively confined to the nuclear compartment, where they are constitutively bound to chromatin structures [28].

#### 1.3.2. The Skin as an Independent Peripheral Endocrine Organ

The progressive characterization of the skin has clarified that this district fulfils the functional requirements to be considered as an independent peripheral endocrine organ. Indeed, recent evidence indicates that the skin is actively involved in the synthesis and metabolism of several hormones, including sex steroids, vitamin D, calcitriol and insulin-like growth factor (IGF) [1,4]. 

Steroidogenesis is the process involved in the conversion of cholesterol into active steroid hormones that can be classified into glucocorticoids, mineralocorticoids and sex steroids, the latter including androgens, estrogens and progestogens. This process occurs primarily in endocrine organs such as the adrenal gland and the gonads though it can also prevail in human skin. Specifically, the cutaneous district expresses all the major enzyme systems that are required for the de novo synthesis of androgens [29,30]. 

Human skin is a relevant source of vitamin D, being the sole site involved in the synthesis of cholecalciferol. This hormone is known to regulate skin homeostasis by mediating the proliferation and differentiation of the epidermis and epidermal appendages [31]. Moreover, vitamin D contributes to the regulation of cutaneous immune responses by counteracting inflammation [32]. 

In addition, dermal fibroblasts and melanocytes can synthesize IGF-1, while epidermal keratinocytes, melanocytes, hair follicles and endothelial cells are shown to produce adrenocorticotropic hormone (ACTH) and the melanocyte-stimulating hormone (MSH) [1].

The metabolism of hormones, aimed either at the activation or inactivation of these chemical messengers, is known to occur in human skin through complex mechanisms that require strict functional coordination amongst different cutaneous cellular populations and yield pathways primarily involved in the transformation of sex steroids, retinoids and vitamin D [27,33]. 

In particular, the metabolism of sex hormones includes the hydrolysis of dehydroepiandrosterone (DHEA)-sulfate, a compound produced in the adrenal glands that is then released in the circulation, into DHEA through the enzymatic activity of the steroid sulfatases expressed by sebocytes and the cells of the dermal papilla [1,30]. Next, DHEA is converted to androstenedione by the 3β-hydroxysteroid dehydrogenase⁄∆-isomerase (∆5-3β-HSD) in the sebaceous gland and is then finally transformed into testosterone by the 17β-hydroxysteroid dehydrogenase (17β-HSD) enzyme in the pilosebaceous unit and in epidermal keratinocytes [34].

Skin cells are also able to convert testosterone into the more active metabolite 5α-dihydrotestosterone (DHT) in a reaction catalyzed by 5-α-reductase, an enzyme highly expressed in keratinocytes [34]. Moreover, the transformation of testosterone into 17β-estradiol (E2) and of androstenedione into estrone (E1) is mediated by the CYP19 aromatase that is highly expressed in the skin district. In addition, E1 can be metabolized into E2 by the 17β-HSD enzyme that is greatly expressed in different skin cells [35]. Finally, most skin cellular populations can inactivate estrogens by sulfation [35].

#### 1.3.3. Role of Sex Hormones on Human Skin

Sex steroids, herein referred to as sex hormones, are represented by androgens, estrogens and progestogens and are molecules presenting multiple effects on human skin. Their function is exerted following their binding to intracellular cytoplasmic hormone receptors that, upon activation, translocate to the nucleus and act as transcriptional regulators for several target genes [4,34].

Androgens receptors (ARs) are expressed in the cytoplasm of different skin cell types, including keratinocytes, sebocytes, fibroblasts, endothelial cells, sweat gland cells and cells of the dermal papilla. The binding of androgens to ARs is known to induce several effects on various skin cell populations, such as: the regulation of the function of the sebaceous glands by controlling the proliferation of sebocytes and sebum secretion [34]; the modulation of hair growth by acting on the cells of the dermal papilla [36]; the regulation of inflammatory responses [37]. In particular, androgens are primarily regarded as hormones exerting anti-inflammatory functions by inducing a direct attenuation of the inflammatory process in immune cells that express high levels of ARs such as keratinocytes, macrophages, neutrophils, monocytes and T-cells. Direct suppressive effects of androgens on inflammation are induced following the activation of diverse and overlapping signalling routes, principally involving the ERK-1 or ERK-2 pathway, that ultimately modulate the expression and the activity of inflammatory mediators, including TNF-α, IL-1β and the C reactive protein [37,38].

The specific role of androgens on keratinocyte function is still controversial and under investigation. It was shown that testosterone stimulates keratinocyte proliferation during wound healing and epidermal barrier formation [38]; nevertheless, intriguingly, in vitro experiments indicate the negative effect of androgens on keratinocyte migration, while on the contrary, in vivo studies highlight an enhancement in the migration process. It is possible to speculate that positive effects on migration require the interaction between keratinocytes and other skin cell populations, such as fibroblasts [39].

Intracellular estrogens receptors (ERs) exist in two isoforms, namely ER-α and ER-β, that are both known to be expressed in various skin cells [40]. Estrogens are involved in the regulation of several aspects related to skin function comprising cutaneous pigmentation, aging, proliferation, wound healing and inflammation. Interestingly, in terms of inflammatory responses, estrogens mediate both pro-inflammatory and anti-inflammatory functions, an outcome that is strictly dependent on the type of immune cell mediating the response and on the isoform of ER involved in hormonal recognition. In general, the pro-inflammatory activity of estrogens is linked to the induction of the expression of pro-inflammatory mediators as a direct consequence of the activation of the Akt/mTOR signalling route [35,41]. Conversely, estrogens exhibit anti-inflammatory activities primarily by suppressing the NF-κB transduction route [34,35,41]. Other relevant activities exerted by estrogens on human skin are: the regulation of the thickness of the skin and collagen deposition; the promotion of wound healing following the stimulation of dermal fibroblasts by estradiol [40]; the promotion of wound healing by E2 that induces the proliferation of basal epidermal keratinocytes via the Erk/Akt route [42]; the inactivation of estrogens by the sulfotransferase enzyme expressed in keratinocytes of the suprabasal layers, hence promoting keratinocyte differentiation and limiting cellular proliferation [43]. 

**Figure 4:**
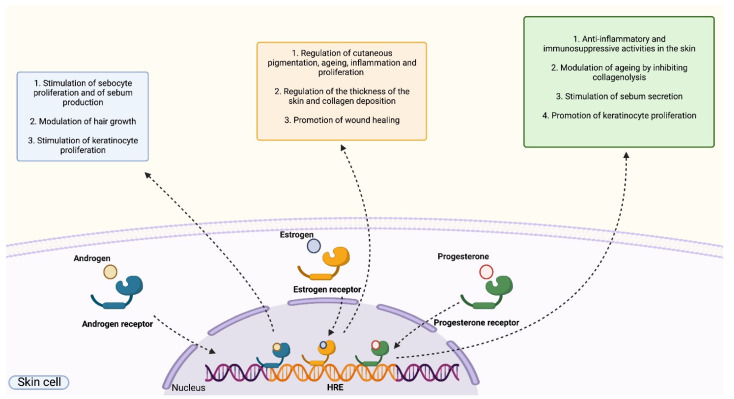
**Role of sex hormones on human skin.** Sex hormones (androgens, estrogens and progesterone) bind to cytoplasmic hormone receptors that act as transcriptional regulators following the recognition of specific DNA sequences, namely hormone-responsive elements (HRE). The binding of androgens to androgen receptors promotes the proliferation of sebocytes and the production of sebum, induce the modulation of hair growth and stimulate the proliferation of keratinocytes. The binding of estrogens to estrogen receptors regulates cutaneous pigmentation, aging, inflammation, proliferation, wound healing, cutaneous thickness and collagen deposition. The binding of progesterone to progesterone receptors exert anti-inflammatory and immunosuppressive activities in the skin, modulating aging by inhibiting collagenolysis, stimulating sebum secretion and promoting keratinocyte proliferation.

Progesterone receptors (PRs) are present in two isoforms, PRA and PRB. PRA possesses nuclear localization, while PRB shows both nuclear and cytoplasmic distribution [44]. Progesterone primarily exerts anti-inflammatory and immunosuppressive activities in the skin since this hormone can suppress the production of cytokines primarily by repressing the NF-κB and MAPK signalling routes as observed both in human and animal models [45,46,47]. Furthermore, as in the case of estrogens, progesterone impacts skin aging through its ability to inhibit collagenolysis and regulate the dermal amount of collagen. Progesterone also acts on sebocytes by stimulating sebum secretion [48] and on keratinocytes by promoting cellular proliferation and by exerting anti-androgenic properties aimed at inhibiting the activity of the 5α-reductase enzyme [49] (Figure 4).

## 2. Inflammatory Skin Diseases with Alterations in Keratinocyte Function and Sex Hormones Imbalance

Considering the role of keratinocytes in skin immunity and the impact of sex hormones on keratinocyte function in terms of regulation of proliferation and differentiation processes, we focus on summarizing the recent findings related to the associations between deregulations in androgens, estrogens and progesterone levels and keratinocyte functions in inflammatory skin disorders.

In order to properly address this issue and delineate an overview on the current information available on this topic, we initially queried the NCBI PubMed database by utilizing the keywords “inflammatory skin disease AND keratinocyte AND (androgen OR estrogen OR progesterone)”. Interestingly, this search allowed the identification of five distinct disorders: Hidradenitis suppurativa (HS); Acne vulgaris (AV); Atopic dermatitis (AD); progesterone hypersensitivity (PH); psoriasis (PSO). 

### 2.1. Hidradenitis Suppurativa 

Hidradenitis suppurativa (HS) is a chronic and debilitating inflammatory follicular occlusive skin disease [50] possessing a broad and heterogenous phenotypic spectrum. HS cases are generally characterized by clinical manifestations, including deep-seated and painful suppurating lesions, nodules, abscesses, tunnels, sinus tracts, fistulas and scars occurring in intertriginous body sites such as the axillae, the anogenital and inguinal regions, the perineum and the infra-mammary folds [7]. The onset of the disease generally occurs in adolescence or early adulthood and affects 1–2% of the population worldwide [7].

Current evidence suggests that the pathogenesis of HS is complex and multifactorial hence showing a strict interplay between different etiologic factors, including genetic background (mutations in *NCSTN*, *PSENEN* and *PSEN1* genes), aberrant immune responses, environmental agents and hormonal imbalance [7]. The major pathogenic feature in HS is shown by the hyperplasia and hyperkeratosis of keratinocytes found in the infundibular region of terminal hair follicles that lead to follicular occlusion and cyst development followed by the subsequent cyst rupture and scattering of its content in the surrounding dermis. These events progressively elicit a deregulated immune activation and a gradual progression towards a chronic inflammatory state [7]. Specifically, these responses seem to exhibit the classical features of a neutrophilic dermatosis characterized by a strong anti-inflammatory response dominated by IL-10 and elicit a clear contribution of TH-17 and TH-1 lymphocytes [7]. 

Of note, growing attention was recently given to the potential role of hormonal imbalance in the aetiology of HS, specifically of sex hormones, therefore strongly suggesting that this aspect needs to be further examined [51]. Indeed, female predominance (3:1 female to male ratio), the post-pubertal onset of the phenotypic manifestations with a peak between 30 and 39 years of age [52], aggravation of clinical symptoms during the premenstrual period and a general improvement of flares during pregnancy and post-menopause, strongly endorse the crucial role played by hormones in the onset, progression and severity of HS [53,54]. 

During the premenstrual period, when the levels of progesterone and estradiol are high, a 44–63% occurrence rate of premenstrual flares is registered in HS women [55]. In addition, females affected by HS are more prone to develop androgen-related sequelae such as hirsutism, acne and irregular menstruation cycles. Moreover, the prevalence of a hyper-androgenic disorder, namely polycystic ovary syndrome (PCOS), amongst HS cases is 9% against the 2.9% registered in non-affected female subjects [56]. While considering therapeutic interventions, different case reports underlined that the administration of progestogen-based oral contraceptives in female cases leads to the onset of exacerbated HS clinical manifestations [51].

Pregnancy seems to significantly impact the phenotype of HS. Although with different final outcomes, many studies indicate an overall improvement of HS flares during pregnancy, when progesterone and oestrogen levels increase, and a progressive worsening after delivery [51].

HS rarely manifests after menopause, and the remission of symptoms generally occurs with increasing age when the production of estrogen and progesterone decreases [51].

Furthermore, the identification of similar phenotypic features occurring between HS and acne vulgaris, the latter is a well known androgen-dependent disorder, strongly suggest that pathogenic patterns involving androgen dysfunction might also exist in HS. Specifically, the principal events considered to support this possible association include the development of premenstrual exacerbations, the onset of most severe forms of the disease in males, and androgen-driven hyperplasia, hyperkeratosis and keratinization of the skin [51,53]. 

Moreover, HS is often associated with obesity, a condition in which enlarged fat cells actively produce hormones [57]. 

Further confirming the importance of hormones in the pathogenesis of HS, Buonomo et al. observed the development or exacerbations of clinical manifestations in two transgender men following the administration of testosterone therapy [58].

Apart from clinical observational studies, some functional assays addressing the possible role of hormones in HS were also conducted. In vivo experiments performed in mouse models showed that androgens induce an incremented pro-inflammatory response driven by augmented levels of TLR-mediated monocyte expression of TNF-α, thus also strongly suggesting a possible correlation between androgenic imbalance and inflammatory phenotypes [59].

In a very recent work, the percentage of AR-positive keratinocytes resulted higher in the epidermis, infundibulum and tunnels of HS lesions when compared to healthy skin. These findings strongly suggest a possible role of AR in follicular occlusion and keratinocyte proliferation [60]. 

Further corroborating these results are microarray analyses that showed enrichment for genes regulated by the activation of AR in lesional areas with respect to nonlesional HS skin partitions. Transcription factors correlated to epidermal stem cells activity were also enriched, and it is known that these cells are localized in the hair bulge that is strongly sensitive to androgens [61]. In another microarray transcriptome study, a gender-specific gene expression profile was observed. Indeed, in apocrine glands derived from lesional skin of HS patients, the upregulation of gene signatures involved in testis differentiation regulated by androgens was detected in female individuals, while the downregulation of genes correlated to lipid metabolism was highlighted in male HS subjects [62].

On the contrary, in an immunohistochemistry study, the levels of expression of ERs and ARs in apocrine glands of HS cases were no different from the controls [63]. 

A functional study conducted on ARs showed a reduced activity of 3 β-hydroxysteroid dehydrogenases and 17 β-hydroxysteroids in apocrine glands obtained from the axilla of HS patients when compared to controls thus indicating a reduced conversion of DHEA and androstenedione [64]. 

When considering serum levels of circulating sex hormones, testosterone and androgen levels showed an increment in HS subjects when compared to controls, although 78% of analyzed cases fell within the normal range [61]. 

An increment in androgen levels was also reported in HS women [65]. Indeed, when individuals in the premenstrual phase were analyzed, patients with HS flares and controls displayed no differences, while HS patients with no flares presented an increment of testosterone, androstenedione and androgen index and a decrement of progesterone levels, though the mechanisms underlining these association still needs to be clarified [52].

Further complicating this already intricate picture is another study that failed in detecting differences in the levels of testosterone, sex hormone-binding globulin and DHEA-sulfate in HS patients and controls matched per age, body mass index and hirsutism. These results suggest possible biases in the previous studies due to confounding factors such as BMI and hirsutism, events that are more predominant in individuals affected by HS [66].

### 2.2. Acne Vulgaris 

Acne vulgaris (AV) is a common chronic inflammatory skin disease affecting approximately 9.4% of the world’s population, exhibiting the highest prevalence in adolescents. AV primarily affects the pilosebaceous unit, hair shaft and sebaceous glands and is characterized by the development of comedones and inflammatory lesions such as papules, nodules and pustules on the face, neck, trunk, or in the proximal upper extremities of the body [67,68]. 

The pathogenesis of AV is complex and involves different processes, including alterations in sebaceous gland function correlated to increased sebum production (hyperseborrhea) and abnormal sebum composition, hormonal imbalances, follicular hyperproliferation and hyperkeratinization resulting in follicular plugging and rupture and cutaneous dysbiosis associated with the proliferation of *Propionibacterium acnes* in the pilosebaceous unit [8,68,69]. The combination of these events progressively leads to an enhanced cutaneous inflammatory response, mainly driven by an augmented IL-1 production that promotes the development of AV lesions.

To date, the role exploited by androgens in the pathogenesis of AV is well established; indeed, AV is commonly defined as a hyper-androgenic disorder in which DHT, DHEA-sulfate and testosterone levels are found to be higher in patients when compared to healthy controls [69]. 

Sebaceous glands constitute the principal site of the skin compartment involved in steroidogenesis. Sebocytes are known to express the central enzymes required for the conversion of inactive or less active circulating androgens to potent hormone-derivates that, upon binding to intracellular receptors, trigger the transcriptional activation of downstream target genes [70]. Enhanced activation of androgens in AV promotes the proliferation and differentiation of sebocytes and induces lipid synthesis resulting in hyperseborrhea. Lipogenesis is promoted by the AR-dependent activation of mTOR, while the proliferation and differentiation of sebocytes are induced by the suppression of Wnt/Beta-catenin signalling pathway. The combination of these two effects driven by androgens on sebocytes promotes an increment in lipid synthesis and an augmented release of sebum in the sebaceous duct [71]. In addition, androgens, together with an altered composition of sebum and high levels of IL-1 pro-inflammatory cytokine, dramatically impact keratinocyte functions by excessively stimulating cellular proliferation and by impairing both differentiation programs and the desquamation of follicular keratinocytes that result in hyperkeratosis and the plugging of the gland’s duct [71].

In a study conducted by Kumtornrut et al., a correlation between androgens, keratinocytes and dermal fibroblast in AV emerged. Dermal fibroblasts express significant levels of ARs, and in vitro analysis showed that growth factors released by these cells upon hormone stimulation could suppress keratinocyte differentiation, thus demonstrating a possible indirect effect of androgens on keratinocytes [72]. 

### 2.3. Atopic Dermatitis

Atopic dermatitis (AD) is a chronic, relapsing and remitting inflammatory skin disease. In its acute phase, AD is clinically characterized by cutaneous pruritic and erythematous lesions, while red or brown flares with cracked or squamous skin appear during the chronic state that is also associated with the development of lichenification and prurigo nodules [73]. AD affects 1–10% of adults and 15–25% of children, bearing an onset below 5 years of age in 80–90% of reported cases [73].

AD presents a multifactorial aetiology accounting for genetic variations, immune dysregulation and environmental factors. AD patients encompass epidermal barrier dysfunctions linked to altered epidermal differentiation processes that are commonly associated with mutations in filaggrin (*FLG*) gene encoding for a structural protein of the skin [74] or correlated to alterations in lipid metabolism that collectively leads to transepidermal water loss and the penetration of external factors through the skin [75]. Immune alterations also play an important role in the pathogenesis of AD and include an excessive activation and massive infiltration of TH2 cells and TH22 lymphocytes in acute lesions, while the TH1 population prevail in the chronic status [76].

The possible role of sex hormones in the development of AD is poorly explored, though some possible associations have been recently unveiled. Evidence suggests that sex hormones might have an impact on the onset and progression of AD by altering cutaneous immune responses and skin permeability functions. 

AD is more frequent in males with respect to females during childhood, but after puberty, this trend is inverted. While considering all ages, intrinsic AD (characterized by normal IgE levels, lack of IgE antibodies against allergens, no epithelial barrier disruption and no *FLG* mutations) is generally more common in females [77]. The high prevalence of AD in young boys might be correlated to lower levels of production of steroid sulfatase, an enzyme responsible for the metabolism of DHEA [78]. During the development phase, the levels of sex hormones rise, and the influence of DHEA should therefore be minor, hence favoring the predominant effect of androgens in suppressing the activity of TH2 cells [78]. Indeed, DHEA and testosterone serum concentrations are lower in male AD individuals when compared to controls, leading to reduced hormonal suppressive effects on immunity and therefore to an aggravation of AD phenotype [79]. Conversely, the levels of DHEA, dihydrotestosterone, testosterone, and follicle-stimulating hormone (FSH) in AD females show no differences when compared to healthy female controls [79].

On the other hand, an impairment in skin barrier function is more pronounced in males, a condition that is worsened by androgens, when compared to females in which higher levels of estrogens are able to promote the reconstitution of the skin barrier [78]. 

Nevertheless, females experience a worsening in the clinical manifestations of AD immediately before menstruation [80]. During the luteal phase of the menstrual cycle, a negative perturbative effect driven by a low estrogen/progesterone ratio predominates in the skin and negatively impacts cutaneous barrier functions by increasing skin permeability, therefore, enhancing the susceptibility towards allergens and irritants. Moreover, increased progesterone and estrogen levels during the premenstrual phase alter the activity of TH2 cells that exacerbate AD conditions by promoting inflammation and IgE production [81]. It was also observed that AD symptoms worsen during pregnancy in 50–60% of analyzed cases, the effect probably due to the relevant increment of estradiol and progesterone that directly impacts the activity of the TH2 cells and the functionality of the skin [82]. 

Intriguingly, the expression of two major regulators of steroidogenesis, namely the steroidogenic acute regulatory protein (StAR) that is a rate-determining protein for steroid synthesis and the metastatic lymph node 64 (MLN64) that is a cholesterol transport protein, were found to be altered in skin biopsies derived from AD patients when compared to healthy controls. Specifically, in AD cases, the StaR protein that is normally expressed in the basal layer was found to be completely absent. Similarly, the production of MLN64 in the suprabasal layer was found to be reduced [83].

### 2.4. Progesterone Hypersensitivity

Progesterone hypersensitivity (PH), also known as autoimmune progesterone dermatitis, is a very rare inflammatory skin disease that occurs periodically in correspondence with the peak of progesterone production during the menstrual period. PH is characterized by acute symptoms including urticaria erythema, rash and vesiculobullous lesions, as well as by delayed hypersensitivity signs leading to chronic dermatitis [84]. In most cases, symptoms arise concomitantly with the peak of endogenous progesterone during the luteal phase and disappear after the menstruation flow; both features are considered as diagnostic markers for the pathology [85]. Elevated levels of basophils and high levels of mast cell activation associated with IgE production are registered in PH cases [86].

In this context, it is speculated that women develop sensitization to progesterone following its release at a systemic level (e.g., oral contraceptives or hormonal stimulation during in vitro fertilization) [87]. On the other hand, a cross-sensitization with molecules structurally similar to progesterone is hypothesized [88]. Alterations in enzymes involved in the cutaneous metabolism of steroid hormones could also account for the development of PH [89]. In addition, some women experienced an exacerbation of the condition during pregnancy, but PH can also arise intrapartum or in the postpartum period, thus suggesting a sensitization due to the high level of progesterone during pregnancy [84].

### 2.5. Psoriasis 

Psoriasis (PSO) is a chronic inflammatory skin disease given by recurrent inflammatory episodes that lead to an impairment in the proliferation and differentiation of keratinocytes. PSO exhibit primary cutaneous manifestations together with a relevant genetic predisposition and possess a worldwide prevalence of about 2% [90].

PSO is characterized by lesions called plaques and papules, displaying epidermal hyperplasia and infiltration of immune cells such as dendritic cells, T-cells, neutrophils and macrophages. The initial phase in the development of the psoriatic lesion involves the keratinocytes of the outermost skin layer that, upon activation triggered by pathogens or mechanical damages, start to interact with other cell types of the deeper layers resulting in the activation of innate immunity mechanisms followed by the onset of the adaptive immune response. Subsequently, the perpetuation of an inflammatory environment promotes the chronicity of the disease [90]. 

In women affected by PSO, menstruation, pregnancy and menopause influence the progress of the disorder, hence suggesting a possible role of sexual hormones on the pathogenesis of the disease. It was observed that estrogens, mainly E2, affect the severity of the disorder by directly impacting inflammation and keratinocyte function as well as angiogenesis and oxidative stress responses [91]. In the pathogenesis of PSO, oxidative stress is found to support inflammatory responses. It was observed that estrogens present anti-oxidative effects and that they positively regulate the expression of the vascular endothelial growth factor (VEGF), collectively resulting in increased survival, proliferation and migration rates of endothelial cells, all of which are histological features typical of psoriatic lesions [91]. 

TH1 and TH17 cells are upregulated in psoriatic plaques, sites in which they release inflammatory cytokines that stimulate inflammatory responses mediated by keratinocytes. Estrogens can suppress the activity of pro-inflammatory cytokines, as well as stimulate TH2 cells to produce anti-inflammatory molecules. Moreover, estrogens promote T-cell conversion into T-regulatory cells, a population actively involved in the modulation of immune responses. In this context, estrogens attenuate PSO, as observed in women during pregnancy [91].

Moreover, in PSO, estrogens tightly affect keratinocyte functions. Indeed, several studies demonstrated that E2 promotes keratinocyte proliferation and migration by inducing the expression of cyclin D2 [92,93]. These findings suggest that estrogens may increase epidermal hyperplasia and consequently worsen the manifestation of the psoriatic lesion. On the other hand, estrogens induce a reduction in cytokine production by keratinocytes, with consequent mitigation of the inflammatory environment, thus improving the symptoms of the disease. These factors favor the emergence of an intriguing aspect, highlighting that estrogens may have a double effect on keratinocytes either by supporting PSO or by suppressing its development [91]. 

## 3. Conclusions

The complex interactions existing between alterations in functional and immune activities of keratinocytes, cutaneous immune dysregulation and endocrine alterations should be further investigated since they might be associated with several immune-mediated skin diseases [5]. Collectively, this work reviewed the current knowledge regarding the role exerted by sex hormones on keratinocyte function in five inflammatory skin diseases: Hidradenitis suppurativa (HS); Acne vulgaris (AV); Atopic dermatitis (AD); progesterone hypersensitivity (PH); psoriasis (PSO). 

The levels of progesterone and estrogens during the menstrual period, pregnancy and menopause highly influence the onset, progression and severity of flares in women affected by HS, AD, PH and PSO. In HS cases, high levels of progesterone and estrogen give rise to an exacerbation of flares during the premenstrual period and an amelioration of symptoms during pregnancy. Conversely, a decrement in estrogen and progesterone during menopause is associated with a remission of symptoms. In AD, the low cutaneous progesterone/estrogen ratio during the luteal phase negatively impacts cutaneous barrier functions by increasing skin permeability, and similar effects were detected during the luteal phase in PH cases. Increased progesterone and estrogen levels during the premenstrual phase and pregnancy exacerbate AD conditions by promoting inflammation and IgE production. Interestingly, in women affected by PSO, progesterone and estrogen appear to both aggravate symptoms by promoting keratinocyte proliferation and migration and reducing cytokine production by keratinocytes hence limiting cutaneous inflammation.

**Figure 5:**
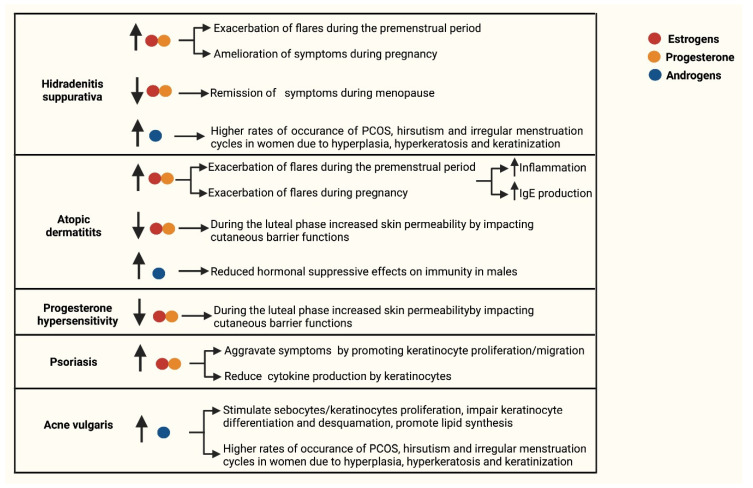
Schematic representation reporting the association between the levels of sex hormones and keratinocyte functions in inflammatory skin disorders. The role of sex hormones, keratinocyte functions and cutaneous immunity in Hidradenitis suppurativa, Atopic dermatitis, progesterone hypersensitivity, psoriasis and Acne vulgaris. In the indicated processes, the arrows facing upwards (↑) indicate an upregulation, while the arrows facing downwards (↓) indicate a downregulation. Estrogen is indicated with a red dot, progesterone by an orange dot and androgens by a blue dot.

Androgenic imbalances are registered in women affected both by HS and AV, disorders in which hyperandrogenisms are associated with higher rates of occurrence of PCOS, hirsutism and irregular menstruation cycles. In addition, in HS lesions, an enrichment for genes regulated by a deregulated activation of ARs was observed and presumably favors proliferation and differentiation programs in keratinocytes. In AV affected cases, high levels of circulating androgens excessively stimulate the proliferation of sebocytes and keratinocytes, impair keratinocyte differentiation and desquamation and also promote lipid synthesis by sebocytes hence resulting in hyperseborrhea. Finally, in AD affected males, low serum concentrations of androgens, in particular of DHEA and testosterone, lead to reduced hormonal suppressive effects on immunity and therefore to an aggravation of AD phenotype (Figure 5). 

The study of these complex cross-talks gave rise to crucial similarities and contradictions amongst the different analyzed disorders. We are convinced that the application of multi-OMICS studies on large groups of clinically well characterized patients is envisaged in order to characterize these complex interactions and to clarify the possible associations between hormonal imbalances and clinical features. Furthermore, the integration of clinical and OMICs findings might provide a better-defined answer relative to the mechanisms involved in HS, AV, AD, PH and PSO, therefore giving novel insights for the definition of tailored therapeutic interventions.

## Data Availability

Not applicable.
